# Microbiota and metabolome responses in the cecum and serum of broiler chickens fed with plant essential oils or virginiamycin

**DOI:** 10.1038/s41598-020-60135-x

**Published:** 2020-03-25

**Authors:** Yan Chen, Jun Wang, Longfei Yu, Tianyue Xu, Nianhua Zhu

**Affiliations:** Key Laboratory of Animal Nutrition, College of animal science and technology, Jiangxi Agricultural University, Nanchang, Jiangxi 330045 P. R. of China

**Keywords:** Microbiology, Microbial communities

## Abstract

This study investigated the cecal microbiota and serum metabolite profile of chickens fed with plant essential oils (PEO) or virginiamycin (VIRG) using high-throughput 16S rRNA gene sequencing and untargeted metabolomics approach. The main aim of this work was to explore the biochemical mechanisms involved in the improved growth performance of antibiotics and their alternatives in animal production. The results showed that both PEO and VIRG treatment significantly increased the relative abundance of phyla Bacteroidetes and decreased the abundance of phyla Firmicutes and genus of *Lactobacillus* in cecal microbiota of chickens. Compared to the control group (CT group), the relative abundance of genus of *Alistipes*, unclassified Rikenellaceae, *Roseburia*, and *Anaeroplasma* was enriched in the PEO group; that of genus *Bacteroides*, *Lachnospiraceae*, and unclassified Enterobacteriaceae was enriched in the cecal microbiota of the VIRG group. Untargeted metabolomics analyses revealed that the PEO treatment modified 102 metabolites and 3 KEGG pathways (primary bile acid biosynthesis and phenylalanine metabolism) in the cecal microbiota, and 81 metabolites and relevant KEGG pathways (fructose and mannose metabolism, biosynthesis of unsaturated fatty acids, and linoleic acid.) in the serum of the chicken. Compared to the CT group, VIRG treatment group differed 217 metabolites and 10 KEGG pathways in cecal contents and 142 metabolites and 7 KEGG pathways in serum of chickens. Pearson’s correlation analysis showed that phyla Bacteroidetes and genus of *Bacteroides, Alistipes*, and unclassified Rikenellaceae (in the VIRG and PE group) were positively correlated with many lipid metabolites. However, phyla Firmicutes and genera *Lactobacillus* (higher in the CT group) were negatively correlated with the lipid and thymine metabolism, and positively correlated with hydroxyisocaproic acid, cytosine, and taurine. This study shows that dietary supplementation with PEO and VIRG altered the composition and metabolism profile of the cecal microbiota, modified the serum metabolism profile.

## Introduction

The intestinal microbiota, the population of microorganisms that inhabit the intestine, plays an important role in the intestinal morphology, immunity, nutrient digestion and absorption, and host health^1–3^. Many studies have demonstrated that intestinal microbiota participates in many metabolic pathways, such as lipid metabolism and amino acid synthesis^4,5^. The mechanism by which PEO promote growth of may be alter gut microflora and hence improved absorption of nutrients^6^, increase absorption of micronutrients in the small intestine^7^ and reducing the deleterious effects of the microbial metabolites^8–10^. There are many alternatives to antibiotics, such as acidifiers, probiotics, oligosaccharides, and plant extracts, which play a growth promoting role by regulating gut microbes in pig and poultry^11^.

Plant essential oils (PEO), which can be extracted from plants by steam distillation, extrusion, or solvent extraction^12,13^, serve as alternatives for antibiotics used in feed for their safety and limited residual effects^5,13–15^. Many studies have shown that PEO can decrease the number of *E. coli* and increase the amount of Lactobacilli in the intestine of broilers^5,16–18^. This has beneficial effects on the intestinal morphology and barrier, and the antioxidant in animals^19^. Also, Altop *et al*. (2018) reported the interactions of host gut–microbiota and co-metabolism on chickens that were fed with essential oils^20^. However, only a few studies have investigated the effects of PEO or antibiotics on the cecum microbiota and serum metabolites profile of the chicken.

In this study, the cecal microbial composition and metabolites in the cecum and serum of chickens that were fed with virginiaymicin (VIRG) or PEO were investigated through 16S rRNA gene sequencing and untargeted mass spectrometry (UPLC-Q-TOF/MS) metabolomic analysis. Comparing the differences in the cecal microbial composition and the potential chemical metabolites might help us understand how the dietary antibiotics or PEO alter the intestinal metabolism and health of the broiler chickens.

## Results

### Diversity and structure of the cecal microbiota

High-throughput sequencing obtained 176,636 quality-controlled reads from 18 cecum samples from three treatments (PEO group,VIRG group and CT group). After denoising, removing chimeras, and filtering low quality sequences, the sample had an average of 44,120 sequences, the average length of each sequence being 443 nucleotides. Based on the 97% identity level, these sequences were decomposed into 1,971 operational taxonomic units (OTUs). The 16S rRNA gene amplicon sequencing results were deposited in the Sequence Read Archive of the NCBI (accession number PRJNA553851).

**Figure 1:**
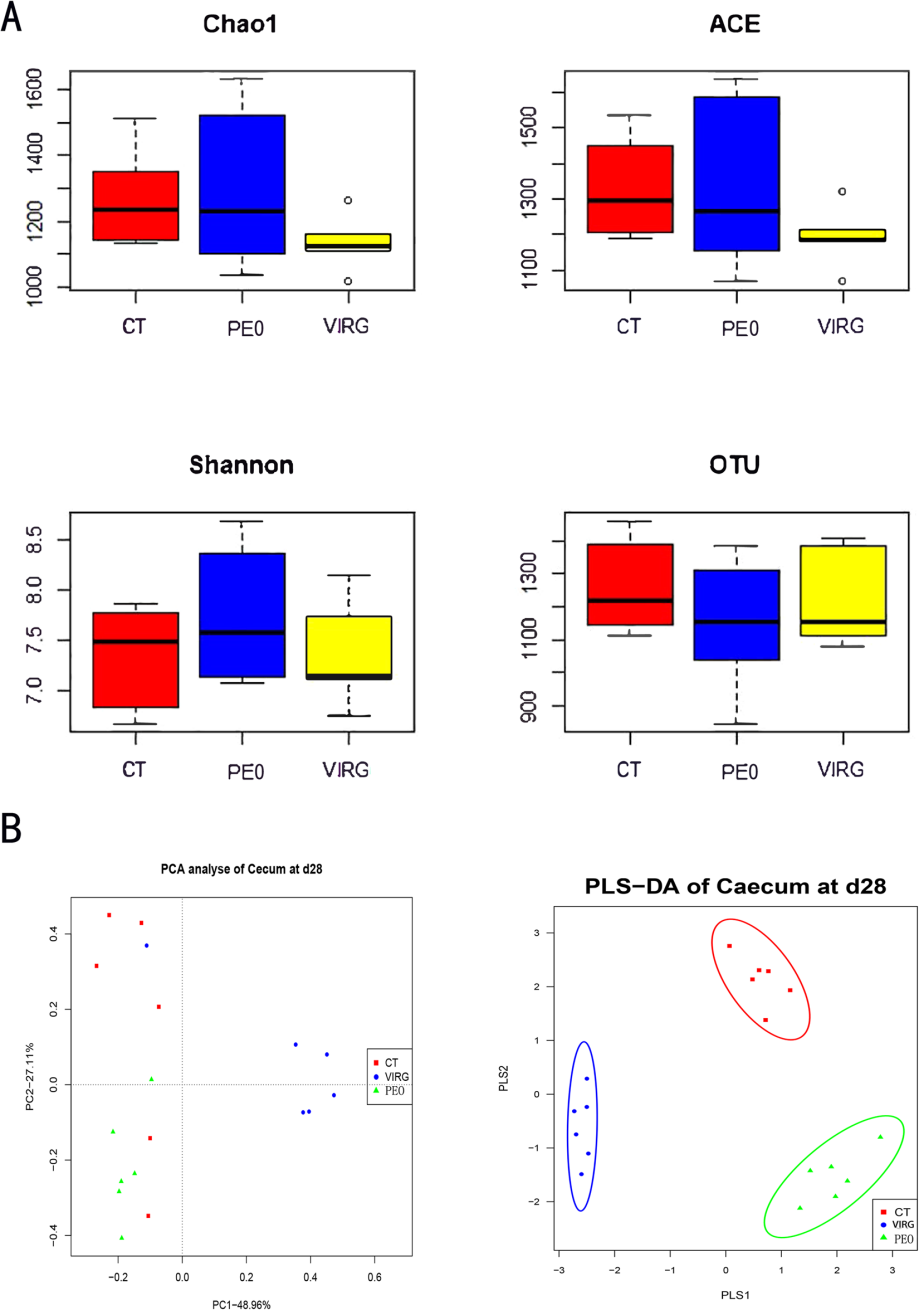
Differences in bacterial community diversity, richness, and structures in the cecum of broiler chickens fed without or with PEO or VIRG. (**A**) Community diversity and richness among CT, PEO, and VIRG group. (**B**) Principal components analysis (PCA) of the bacterial community structure among CT, PEO, and VIRG group. Each symbol represents each gut microbiota. Red symbols represented CT group, blue symbols represented PEO group, and brown symbols represented VIRG group. CT: the basal diet; PEO: the basal diet supplemented with plant extracts; VIRG: the basal diet supplemented with virginiamycin. PLS-DA score plots showed significantly separated clusters between CT, PEO, and VIRG group.

The diversity of the cecal microbiota in the three groups are shown in Fig. 1. Compared to the CT and the PEO group, the Chao1, ACE, and Shannon diversity indices were observed to be significantly lower in the VIRG group. No differences in the diversity indices (Shannon, Chao1, and Observed species) were observed between the PEO and the CT group (Fig. 1A and Supplementary Table 1A). Principal Component Analysis based on EUCLIDEAN distance showed that the cecal microbes, among the three treatments of CT, PEO, and VIRG groups, formed distinct clusters. The treatment groups were well separated with 48.96% and 27.11% variation by the principal components PC1 and PC2, respectively (Fig. 1B). PLS-DA model shows significant separation and discrimination, indicating that the PLS-DA model can be used to identify differences between groups.

**Figure 2:**
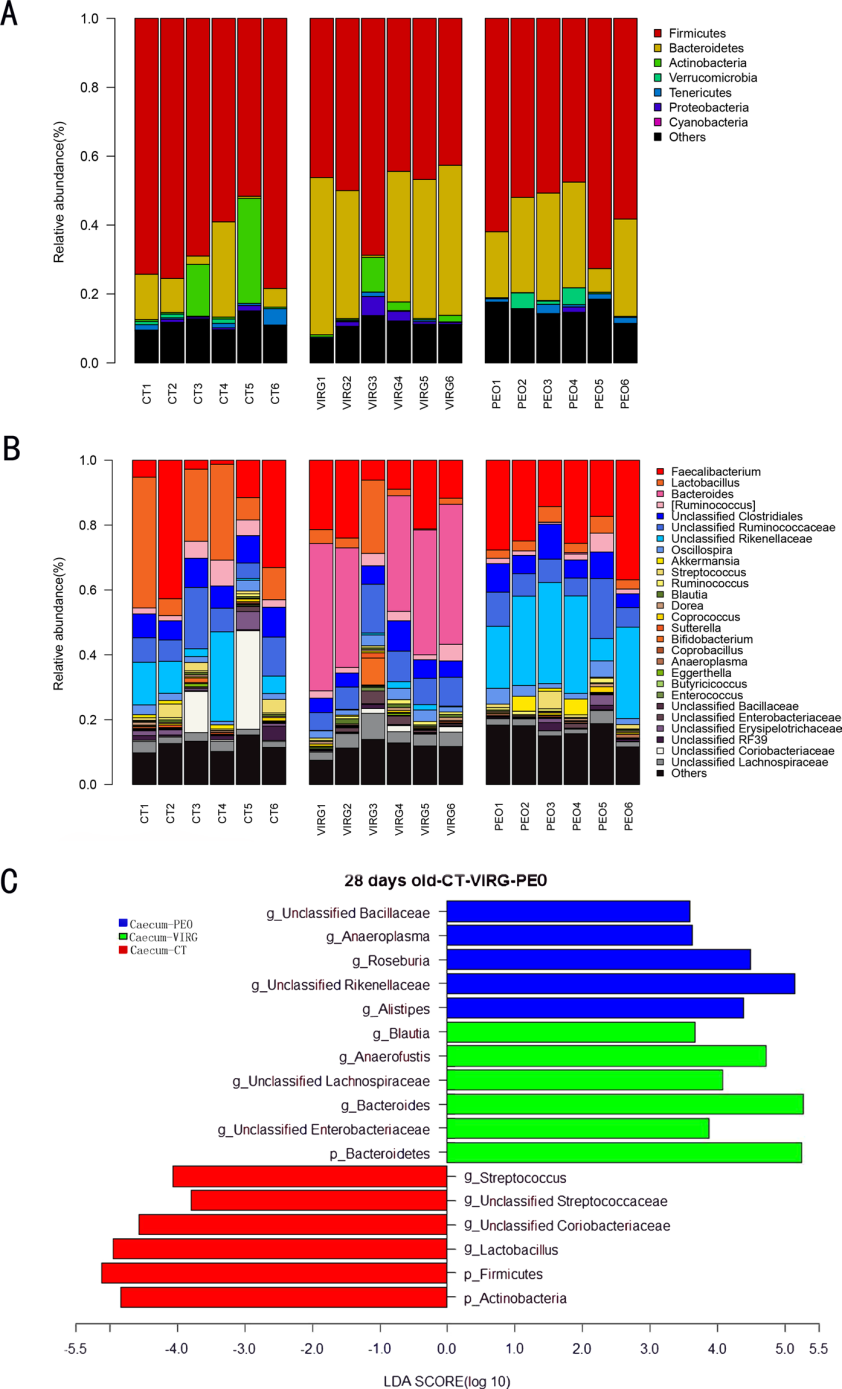
Changes of microbial composition in the cecum of broiler chickens fed without or with PEO or VIRG. Microbial composition at the phylum level (**A**) and genus level (**B**) each bar represents the relative abundance of each bacterial taxa of chicken. Bacterial taxa significantly differentiated between CT, PEO group, and VIRG group (**C**) and was identified by linear discriminant analysis coupled with effect size (LEfSe) using the default parameters. CT: the basal diet; PEO: the basal diet supplemented with plant extracts; VIRG: the basal diet supplemented with virginiamycin.

Dietary treatment with PEO or VIRG (Fig. 2 and Supplementary Table 1B) resulted in a change in the cecal microbial composition. At the phylum level (Fig. 2A), Firmicutes was the most predominant phylum (more than 60%), followed by Bacteroidetes (10–20%), Actinobacteria (2–6%), and Verrucomicrobia (1%). Additionally, there were more than 6% unclassified bacteria in the cecal microbiota of chickens. Compared to the CT group, the relative abundance of phyla Bacteroidetes increased while that of phyla Firmicutes decreased in the cecal microbiota of the PEO and the VIRG groups. At the genus level (Fig. 2B), *Lactobacillus, Faecalibacterium, Bacteroidaceae*, and unclassified *Rikenellaceae* were the predominant genus in the cecal microbiota of the chicken. The relative abundance of *Lactobacillus* was lower, and that of unclassified Rikenellaceae was higher in the cecal microtia of the PEO group. The genus *Bacteroides* was higher in the VIRG group than in the CT group.

The LEfSE test results (Fig. 2, LDA > 2, P < 0.05) showed that the proportion of genera *Alistipes*, unclassified *Rikenellaceae, Anaeroplasma*, unclassified *Bacillaceae*, and *Roseburia* were higher in the cecal microbiota of the PE group (Fig. 2C). The relative abundance of phyla Bacteroidetes and the genus of *Bacteroides*, unclassified *Enterobacteriaceae*, unclassified Lachnospiraceae, and *Anaerofustis* were higher in cecal microbiota of VIRG group. The proportion of phyla Firmicutes and Actionbacteria and genus of *Lactobacillus, streptococcus*, unclassified Erysipelotrichaceae*, coriobacteriaceae*, and *streptococcaceae* were higher in cecal microbiota of CT group (Fig. 2C).

### Effects of dietary plan essential oils or virginiamycin on caecum and serum metabolites

A non-targeted LC–MS-based metabolomics platform was used to analyze the cecum contents and the serum metabolite profiles of chicken fed supplemented with PEO or VIRG. According to the variable importance in the projection (VIP) value > 1, in 95% jack-knifed confidence intervals and P < 0.05, detailed information about the different biomarker metabolites has been shown in Supplementary Table 2. Compared to the CT group, 102 different metabolites with 53 LC-MS/MS(ESI+) and 49 LC-MS/MS(ESI-), were detected in cecum contents of the PEO group; 81 different metabolites (30 ESI+ and 51 ESI-) were detected in the serum of the PEO group. VIRG group have 217 different metabolites (96 ESI+ and 121 ESI-) in cecum contents, and 142 different metabolites (82 ESI+ and 60 ESI-) in the serum as compared to the CT group. There were 305 different metabolites (148 ESI+ and 157 ESI-) in cecum contents, and 119 different metabolites (58 ESI+ and 61 ESI-) in the serum between PEO and VIRG groups.

**Figure 3:**
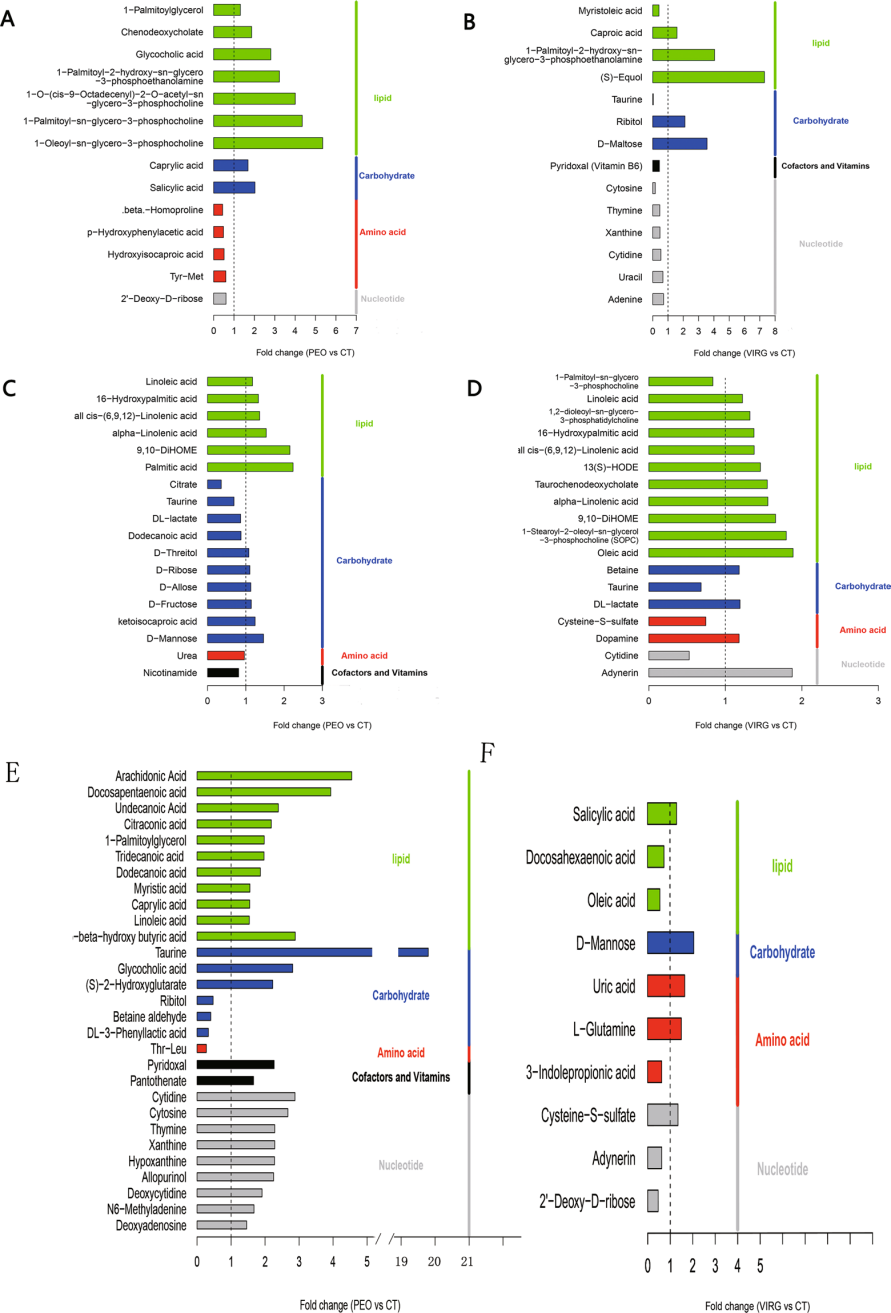
Significantly differential metabolites in the cecum and serum of broiler chickens fed without or with PEO or VIRG. Differential metabolites on PEO vs. CT in the cecum (**A**), VIRG vs. CT and VIRG in the cecum (**B**); differential metabolites on PEO vs. CT in the serum (**C**), VIRG vs. CT and VIRG in the serum (**D**). Metabolites accountable for class discrimination with VIP > 1 and P < 0.05 were listed. CT: the basal diet; PEO: the basal diet supplemented with plant extracts; VIRG: the basal diet supplemented with virginiamycin.

Next, these different metabolites were conclusively identified using the in-house databases. Figure 3 shows the different metabolites in the cecal contents and serum. Compared to the CT group, dietary supplementation with PEO resulted in six more lipid metabolites and two more Carbohydrate metabolites in the cecal contents. These included 1-Oleoyl-sn-glycero-3-phosphocholine (LysoPC(18:1(9Z))), 1-Palmitoyl-2-hydroxy-sn-glycero-3-phosphoethanolamine (LysoPE(16:0/0:0)), 1-Palmitoyl-sn-glycero-3-phosphocholine, Glycocholic acid, Caprylic acid, and Chenodeoxycholate. Amino acid related metabolites (Isoleucyl-valine, Hydroxyproline, beta-Homoproline) decreased in the cecal contents of chicken fed with PEO (Fig. 3A). Additionally, the PEO treatment resulted in 6 more lipid metabolites (Palmitic acid, 9,10-DiHOME, etc.) and six more Carbohydrate metabolites (such as D-Mannose) in the serum of chickens. Compared to the CT group,some metabolites (Citrate, taurine, nicotinamide, and urea) were lower in the serum of PEO group (Fig. 3C). Compared to the VIRG group, PEO group increased 11 lipid metabolites (Arachidonic Acid, docosapentaenoic acid, linoleic acid, 1-Palmitoylglycerol, dodecanoic acid(C12), myristic acid(C14), caprylic acid(C8), β-hydroxy butyric acid (C4), FC > 1.5) and Cofactors and Vitamins (Pyridoxal (VB6) and Pantothenate) in the cecal contents (Fig. 3E).

Compared to the CT group, dietary supplement with virginiamycin increased the levels of (S)-Equol, LysoPE (16:0/0:0), Caproic acid, D-Maltose, and ribitol in the cecum of the chicken. However, 9 other metabolites including nucleotides (Cytosine, Thymine, Ademine, and Uracil), Myristoleic acid, and Pyridoxal (Vitamin B6) decreased (Fig. 3B). About 10 lipid metabolites such as oleic acid, 9,10-DiHOME, 1-Stearoyl-2-oleoyl-sn-glycerol-3-phosphocholine (SOPC), Adynerin, betaine, DL-lactate, and dopamine were higher in the serum of VIRG group. However, four metabolites (1-Palmitoyl-sn-glycero-3-phosphocholine,cytidine, taurine, cysteine-s-sulfate) were lower in the serum of chicken fed with virginiamycin (Fig. 3D). Only 10 metabolites were different in serum of chickens between PEO and VIRG groups (Fig. 3F).

### The relationship of different relative abundance of bacteria in the cecal microbiota with cecal and serum metabolic

**Figure 4:**
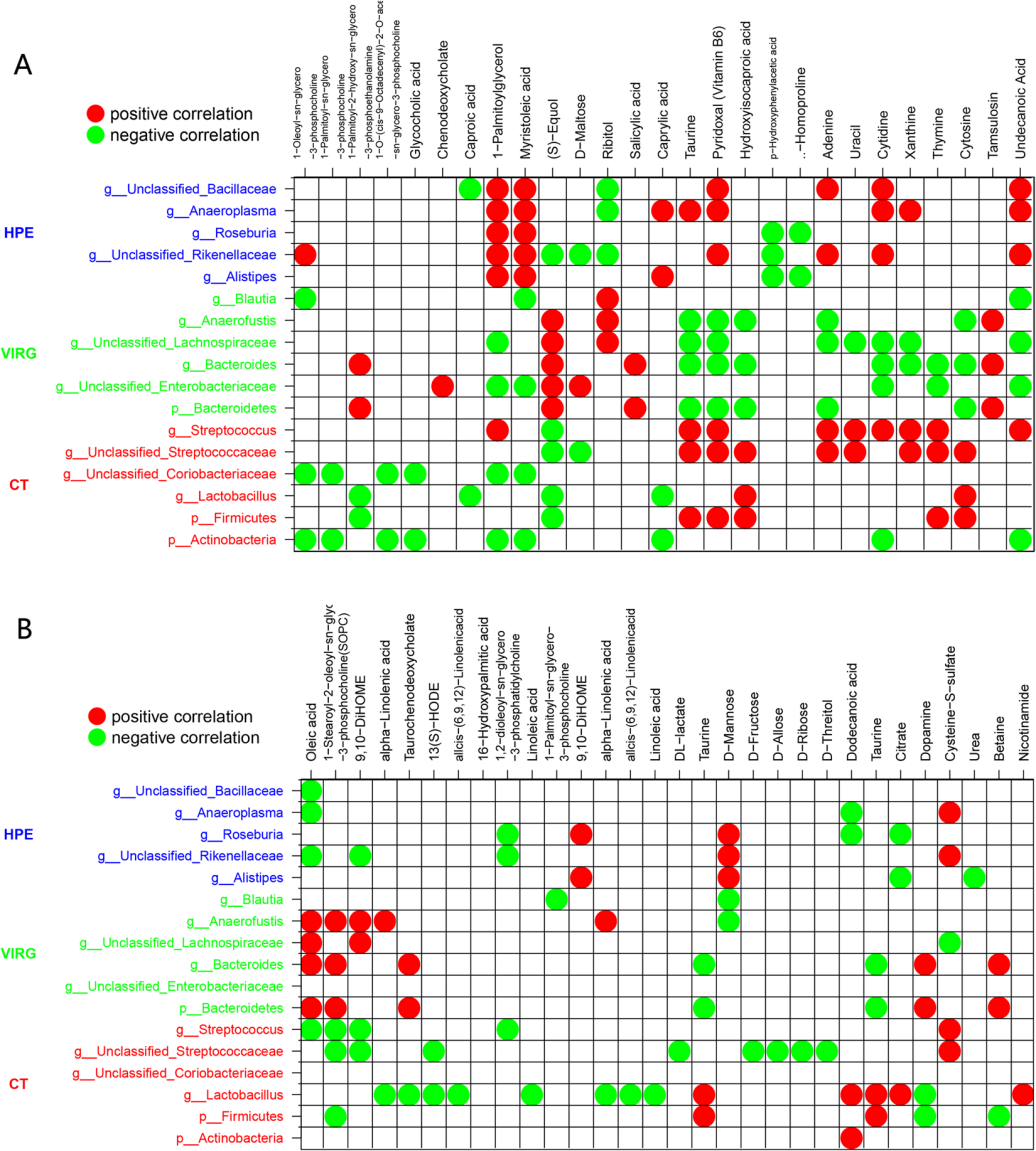
Correlation between microbiota and metabolites in the cecum (**A**) and serum (**B**) of broiler chickens fed without or with dietary PEO or VIRG. The color was according to the Spearman correlation coefficient distribution. Red represented significant positive correlation (P < 0.05), blue represented significantly negative correlation (P < 0.05), and white represented that the correlation was not significant (P > 0.05). CT: the basal diet; PEO: the basal diet supplemented with plant extracts; VIRG: the basal diet supplemented with virginiamycin.

Pearson’s correlation analyses showed that the relative abundance of different bacteria (LEfSE) at the genus and phylum level in the cecal microbiota were found to be closely associated to the concentration of specific microbial metabolites in the cecum and serum of chickens (Fig. 4). The high proportion of bacteria in the cecal microbiota of the PEO group was positively correlated with 1-Palmitoylglycerol, myristoleic acid, and Pyridoxal (Vitamin B6) in the cecum and D-Mannose in the serum. Additionally, the relative abundance of unclassified *Rikenellaceae* positively correlated with 1-Oleoyl-sn-glycero-3-phosphocholine, adenine, cytidine, and undecanoic acid in cecum (Fig. 4A) and negatively correlated with (S)-Equol, p-Hydroxyphenylacetic acid, and ribitol in the cecum and in the serum of chicken (Fig. 4B). The genus *Alistipes* positively correlated with 1-Palmitoylglycerol, myristoleic acid, and caprylic acid in the cecum and D-Mannose and 9,10-DiHOME in the serum of chicken, negatively correlated with β-Homoproline and p-Hydroxyphenylacetic acid in caecum and in citrate and urea in serum.

The high proportion of bacteria in the cecal microbiota of the VIRG group positively correlated with ribitol and (S)-Equol in the cecum and oleic acid, SOPC, and adynerin in the serum, and negatively correlated with cytidine, hydroxyisocaproic acid, and Pyridoxal (Vitamin B6) in the cecum. Additionally, the relative abundance of phylum Bacteroidetes and genera *Bacteroides* positively correlated with LysoPE(16:0/0:0), salicylic acid, and tamsulosin in the cecum and betaine, taurochenodeoxycholate, and dopamine in the serum while negatively correlating with hydroxyisocaproic acid, cytosine, taurine, and Pyridoxal (Vitamin B6) in the cecum and taurine in the serum. The relative abundance of phylum Firmicutes positively correlated with hydroxyisocaproic acid, cytosine, taurine, and Pyridoxal (Vitamin B6) in the cecum and taurine in the serum, while negatively correlating with LysoPE(16:0/0:0) and (S)-Equol in the cecum and SOPC, betaine, and dopamine in the serum. The relative abundance of genera *Lactobacillus* positively correlated with hydroxyisocaproic acid and cytosine in the cecum and taurine, dodecanoic acid, citrate, and nicotinamide in the serum, while negatively correlating with LysoPE(16:0/0:0), (S)-Equol, and caprylic acid in the cecum and alpha-linolenic acid, cis-(6,9,12)-linolenic acid, linoleic acid, taurochenodeoxycholate, 13(S)-HODE, and dopamine in the serum.

### Effects of dietary plan essential oils or virginiamycin on KEGG pathway in caecum and serum

**Figure 5:**
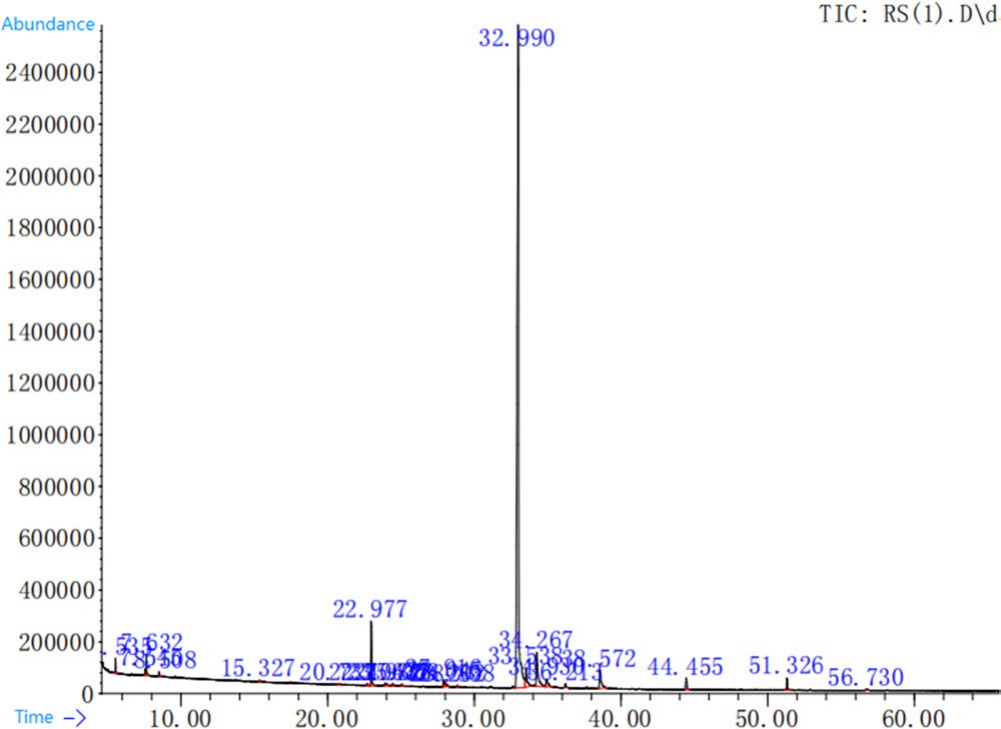
Gas chromatography-mass spectrometry total ion chromatogram of PEO. Peaks are representative of the mass of the compounds present in PEO: Cinnamical dehyde (RT = 33.6, Area = 78.3%), Isophorone (RT = 22.9, Area = 4.23%).

Based on these metabolites (VIP > 0.1, P < 0.05), the software Metaboanalyst 3.0 was used to enrich the relevant KEGG pathways. The relevant KEGG pathways enriched by metabolites between PEO within the CT group are shown in Table 1. As compared to the CT group, three relevant KEGG pathways were significantly enriched for these metabolites in the cecum of the PEO group, including cecal primary bile acid biosynthesis (up-regulated by Chenodeoxycholate (1.86 FC (Fold change)) and Glycocholic acid (2.80 FC)); cecal Phenylalanine metabolism (up-regulated by Salicylic acid (2.02 FC) and Pyrimidine metabolism. There were six relevant KEGG pathways enriched in the serum of chickens of the PEO group (Fig. 5C), including fructose and mannose metabolism (up-regulated by D-mannose (1.46 FC) and D-allose (1.13 FC)), biosynthesis of unsaturated fatty acids, Linoleic acid metabolism, and serum fatty acid biosynthesis were enriched.

**Table 1:** The relevant KEGG pathways enriched by metabolites between PEO with CT group.

	Metabolites(cpdName)	Map.Name	Map_ID	Pathway hierarchy
caecum	Chenodeoxycholate(1.86FC^1^), Glycocholic acid (2.80FC)	Primary bile acid biosynthesis	map00120	Lipid metabolism
	p-Hydroxyphenylacetic acid(0.48FC), Salicylic acid(2.02FC)	Phenylalanine metabolism	map00360	Amino acid metabolism
	Thymine(0.63FC), Deoxycytidine (1.62FC)	Pyrimidine metabolism	map00240	Nucleotide metabolism
serum	D-Mannose(1.46FC), D-Allose(1.13FC)	Fructose and mannose metabolism	map00051	Carbohydrate metabolism
	Linoleic acid(1.44FC), alpha-Linolenic acid(1.53FC), all cis-(6, 9, 12)-Linolenic acid(1.36FC), Palmitic acid(2.23FC)	Biosynthesis of unsaturated fatty acids	map01040	Lipid metabolism
	Linoleic acid(1.46FC), all cis-(6, 9, 12)-Linolenic acid(2.23FC)	Linoleic acid metabolism	map00591	
	Dodecanoic acid(0.87FC), Palmitic acid(1.36FC)	Fatty acid biosynthesis	map00061	
	Taurine(0.69FC), D-Mannose(1.46FC), D-Allose(1.13FC), D-Ribose(1.105FC), Urea(0.96FC), Betaine(1.43FC)	ABC transporters	map02010	Membrane transport
	D-Mannose(1.46FC)	Lysosome	map04142	Transport and catabolism

^1^FC = Fold change.

The relevant KEGG pathways enriched by metabolites between VIRG within the CT group are shown in Table 2. Dietary supplement virginiamycin altered 10 cecal KEGG pathways and seven serum KEGG pathways of chicken. D-mannose and L-leucine up-regulated the KEGG pathway of Lysosome and the mTOR signaling pathway respectively. D-glucosamine 6-phosphate up-regulated and L-alanine down-regulated the KEGG pathway of alanine, aspartate, and glutamate metabolism. Many metabolites such as dodecanoic acid, myristic acid, oleic acid, taurine, L-alanine, uracil, pantothenate, and Pyridoxal down-regulated the cecal KEGG pathway of fatty acid biosynthesis, beta-alanine metabolism, taurine and hypotaurine metabolism, pantothenate, and CoA biosynthesis. In serum, lipid metabolism KEGG pathway were enriched in the serum of the chicken feed with VIRG, including biosynthesis of unsaturated fatty acids (up-regulated by oleic acid (1.88 FC), alpha-linolenic acid (1.55 FC), linoleic acid (1.41 FC), all cis-(6,9,12)-linolenic acid (1.37 FC), linoleic acid metabolism (linoleic acid (1.41 FC), all cis-(6,9,12)-linolenic acid (1.37 FC), SOPC (1.79 FC), and alpha-linolenic acid metabolism (alpha-linolenic acid (1.55 FC), SOPC (1.79 FC)). Primary bile acid biosynthesis was increased by Taurochenodeoxycholate (1.547 FC) and decreased by Taurine (0.68 FC). Then, citrate cycle (Citrate (0.68 FC), L-Malic acid (0.83 FC)), and Pyrimidine metabolism (Urea (0.62 FC), Cytidine (0.53 FC), Methylmalonic acid (0.52 FC),) in serum were decreased.

**Table 2:** The relevant KEGG pathways enriched by metabolites between VIRG with CT group.

	Metabolites(cpdName)	Map.Name	Map_ID	Pathway hierarchy
caecum	Thymine(0.46FC^1^), Cytidine(0.53FC), Uracil(0.69FC), Pseudouridine Cytosine(0.17FC),	Pyrimidine metabolism	map00240	Nucleotide metabolism
	D-Maltose(3.55FC), Taurine(0.73FC), L-Leucine L-Alanine(0.54FC), D-Mannose(6.91FC)	ABC transporters	map02010	Membrane transport
	Dodecanoic acid(0.57FC), Myristic acid(0.41FC), Oleic acid (0.46FC)	Fatty acid biosynthesis	map00061	Lipid metabolism
	L-Alanine(0.54FC) D-glucosamine 6-phosphate(1.63FC)	Alanine, aspartate and glutamate metabolism	map00250	Amino acid metabolism
	Taurine(0.73FC), L-Alanine(0.54FC)	Taurine and hypotaurine metabolism	map00430	Metabolism of other amino acids
	Uracil(0.56FC), Pantothenate(0.63FC)	beta-Alanine metabolism	map00410	
	4-Pyridoxic acid(1.68FC), Pyridoxal(0.43FC)	Vitamin B6 metabolism	map00750	Metabolism of cofactors and vitamins
	Uracil(0.56FC), Pantothenate(0.63FC)	Pantothenate and CoA biosynthesis	map00770	
	D-Mannose(6.91FC)	Lysosome	map04142	Transport and catabolism
	L-Leucine(2.34FC)	mTOR signaling pathway	map04150	Signal transduction
serum	Citrate(0.68FC), L-Malic acid(0.83FC)	Citrate cycle (TCA cycle)	map00020	Carbohydrate metabolism
	Oleic acid(1.88FC), alpha-Linolenic acid(1.55FC), Linoleic acid(1.41FC), all cis-(6,9,12)-Linolenic acid(1.37FC)	Biosynthesis of unsaturated fatty acids	map01040	Lipid metabolism
	Linoleic acid(1.41FC), all cis-(6, 9, 12)-Linolenic acid(1.37FC), 1-Stearoyl-2-oleoyl-sn-glycerol 3-phosphocholine (SOPC)(1.79FC),	Linoleic acid metabolism	map00591	
	alpha-Linolenic acid(1.55FC), 1-Stearoyl-2-oleoyl- sn-glycerol 3-phosphocholine (SOPC)(1.79FC)	alpha-Linolenic acid metabolism	map00592	
	Taurine(0.68FC), Taurochenodeoxycholate(1.547FC),	Primary bile acid biosynthesis	map00120	
	Urea(0.62FC), Cytidine(0.53FC), Methylmalonic acid(0.52FC),	Pyrimidine metabolism	map00240	Nucleotide metabolism
	L-Tyrosine(1.17FC)	Melanogenesis	map04916	Endocrine system

^1^FC = Fold change.

## Discussion

Since the ban on antibiotic growth-promotion (AGP) in many countries, several effective alternatives to AGPs have been developed in recent years. One of them, PEO or phytogenic additives are considered as appropriate candidates due to their safety benefits^15,21^. Our previous study has shown that dietary supplementation with PEO improved the growth and feed efficiency in broiler chickens^22^, which was consistent with other studies (reviewed by Windisch *et al*., 2008; Zeng *et al*., 2015; and Diaz-Sanchez *et al*., 2015)^14,23,24^. PEO might promote growth by altering gut microflora and hence improving absorption of nutrients^6^, increasing the absorption of micronutrients in the small intestine^7^, stimulating the production and activity of digestive enzymes (such as trypsin and amylase)^25,26^, and stimulating digestive and physiology metabolism^27^.

This study shows that both PEO and VIRG treatments increased the relative abundance of phyla Bacteroidetes and decreased the relative abundance of phyla Firmicutes and genera of *Lactobacillus* and *streptococcus* in the cecal microbiota of chickens. Meanwhile, the relative abundance of phyla Bacteroidetes and genera *Alistipes, Roseburia*, and unclassified *Rikenellaceae* increased in the cecal microbiota of the PEO group. These finding were consistent with previous studies that reported that PEO decreased the number of *Lactobacilli*^28,29^. Next, some researchers reported that there was a significant increase in the *Lactobacillus* in the intestines of chickens fed with PEO or *Macleaya cordata* extracts^18^. These differences may be due to the difference in the source and breed of PEOs, feeding patterns, and sampling parts of the chicken^14^.

The intestinal microbiota plays multiple roles in the intestinal morphology, immunity, nutrient absorption and metabolism, and host health^1,2^. Phyla Bacteroidetes are gram-negative bacteria that ferment polysaccharides and other indigestible carbohydrates and produce short-chain fatty acids (SCFAs) that are gut-friendly. Bacteroidetes are related to fat accumulation in chickens^30^. *Alistipes* and unclassified Rikenellaceae, which belong to phylum Bacteroidales, are generally considered beneficial to the host gut^31^. *Alistipes* can produce succinic acid and other long-chain fatty acids such as C15^32^. Rikenellaceae can produce propionic and succinic acids by fermentation of glucose, lactose, mannose, and melibiose, and formed the iso-methyl branched-chain fatty acid or long chain saturated acids^33^. Genus *Roseburia* belongs to the *Lachnospiraceae* family, a butyrate-producing organism, with a high capacity to form conjugated linoleic acid from linoleic acid^34^. Our Pearson’s correlation analyses revealed that phylum Bacteroidetes were positively correlated with LysoPE (16:0/0:0) and (s)-equol in the cecum and betaine, taurochenodeoxycholate, and dopamine in the serum, while negatively correlating with hydroxyisocaproic acid, cytosine, and taurine in the cecum and taurine in the serum. Moreover, unclassified Rikenellaceae and *Alistipes* were positively correlated with 1-Palmitoylglycerol, myristoleic acid, and Pyridoxal (Vitamin B6) in the cecum and D-Mannose in the serum. These metabolites participated in many biological functions. e.g. LysoPE (16:0/0:0), a lysophospholipid, that serves important signaling functions. Equol may enhance the actions of soy isoflavones, due to its greater affinity for estrogen receptors and have unique antiandrogenic and antioxidant activities^5^. Dopamine is a major transmitter in the extrapyramidal system of the brain and is important in regulating movement. A family of receptors (dopamine receptors) mediates action, which plays a major role in the reward-motivated behaviour^35^. The enrichment of the relative abundance of Phyla Bacteroidetes and genera *Alistipes*, unclassified Rikenellaceae might be related with biosynthesis of fatty acid and lipid metabolism. Recently, some researchers have reported that the increase in the proportion of Phyla Bacteroidetes is related to promoting animal growth performance. On the contrary, Although Lactobacillus is considered to be a beneficial probiotic to the host intestinal health and growth^36^, our results showed that the proportion of Lactobacillus in the cecum negatively correlated with lipid metabolites, such as LysoPE (16:0/0:0) and (S)-Equol in the cecum and alpha-linolenic acid, all cis-(6,9,12)-linolenic acid, linoleic acid, taurochenodeoxycholate, and dopamine in the serum; and positively correlated with some metabolites which accelerated lipid peroxidation (such as hydroxyisocaproic acid and cytosine), down-regulated KEGG pathway related to primary bile acid biosynthesis and citrate cycle (TCA cycle). These results were consistent with De Boever *et al*.^37^, who reported that *Lactobacillus* impaired lipid absorption and consequently resulted in the dietary energy losses^37^. Recently, some reports showed that some *Lactobacillus* strains had a negative influence on the growth performance^30,38^, some strains of *Lactobacillus* are retailed as weight loss probiotics while others are reported with the ability to reduce obesity for increasing appetite and feed consumption^39,40^. Therefore, alteration of intestinal flora may result to improve of animal performance.

Metabolomic analysis revealed that chenodeoxycholate and glycocholic acid up-regulated the KEGG pathway of primary bile acid biosynthesis in the cecal contents of PEO group, Primary bile acids are also critical to the digestion and absorption of fat^41^. In the blood of chickens of PEO group, six lipid metabolites (such as LysoPC (18:1(9Z)) and LysoPE (16:0/0:0)) enriched fatty acid biosynthesus, Lyso PCs perform essential functions in the lipid metabolism of organisms^9,42^; 1-Palmitoylglycerol is the biosynthetic precursor of phosphatidic acid: the major component of lysophosphatidic acid, which is a pluripotent lipid mediator which controls growth, motility, and differentiation^43^. In addition, D-Mannose up-regulated KEGG pathway of fructose and mannose metabolism and ABC transporters in serum of the PEO group. ABC transporters utilize the energy of ATP binding and hydrolysis to transport various substrates across cellular membranes^44^. Above all, dietary supplementation with PEO alters the cecal and blood metabolic pathways, The biosynthesis pathways of fatty acids and unsaturated fatty acids that are closely related to host lipid metabolism might be an important mechanism for growth promoters of PEO^13^.

The mechanism of improving performance of AGPs is related to reduced incidences of subclinical infections, stability of the microbial ecology, thinning of the intestinal wall, suppression of inflammation, and the reduction of bioamines and toxins produced by the bacteria^45,46^. Recently, extensive research on the effects of antibiotics on intestinal biochemicals has been carried out using culture-independent methods and metabolomic analysis^9,10,47^. Many reports showed that the relative abundance of phyla Bacteroidetes increased and that of genus Lactobacillus decreased in the intestines of chickens after the administration of antibiotic^9,10,47–50^, Higher numbers of lactobacilli were previously implicated in broiler growth depression due to competition in nutrient uptake or impaired fat absorption^30,48^. Some other studies showed that proportion of phyla Firmicutes and genus of *Lactobacillus* increased in the gut of the chicken after administration of antibiotics^51–53^. Metabolomic analysis showed that 4-EOTC treatment improved the synthesis of lysophosphatidylcholine (LysoPC), as indicated by the lipid biomarkers LysoPC (16:0), LysoPC (18:3), LysoPC (20:3), and LysoPC (20:4) in the blood of Wistar rats. VIRG treatment altered 218 biochemicals (156 increased, 62 decreased) in the ileum of chickens, including many long chain saturated and polyunsaturated fatty acids; several lysophospholipids also increased in the process^10^. This observation was corroborated by our data that VIRG treatment increased the relative content of phyla Bacteroidetes and decreased that of genus Lactobacillus in the cecum, enriched lipid metabolites and biosynthesis of fatty acid and linoleic acid, and reduced some metabolites (such as urea) that may be toxic or harmful to growth of host.

Compared to the VIRG group, PEO group also increased the content of D-Mannose and arachidonic acid, docosapentaenoic acid, linoleic acid in the cecum of chickens. These metabolites are essential fatty acids with several biological functions: as the precursor that are metabolized by various enzymes to a wide range of bioactive components for growth and proper health^54^. Arachidonic acid is catalyzed by cyclooxygenase (COX) to generate prostaglandins (PGs), which regulate fever, inflammation, smooth muscle contraction, and translocation of water and salt in the kidney^55,56^. In addition, PEO treatment increases the biosystem of Pyridoxal (VB6) and Pantothenate in the cecal contents. Pyridoxal participates protein and sugar metabolism^57^, and the active form of VB6 is linked with adipogenesis^58^. These results indicate that PEO might alter different lipid metabolites and vitamins from AGP.

In summary, this study investigated the microbial profile and metabolites in the cecum and serum of chickens fed with PEO or VIRG by combined microbiome and metabolomic analysis. Our results demonstrated that PEO treatment increased the relative abundance of phyla Bacteroidetes and genera Alistipes, unclassified Rikenellaceae and decreased that of phyla Firmicutes and genus of Lactobacillus in the cecum. Many Lipid metabolites and KEGG pathway of fatty acid biosynthesis were enriched in cecum and serum of chickens in the PEO and VIRG group. These finding provide better understanding of the mechanism of promoting performance of PEO or VIRG, which could further provide useful information for developing an effective and safe alternative to AGP in poultry industry.

## Materials and Methods

### PEO chemical composition

PEO product purchased from Guangdong Ruisheng Technology Co., Ltd (Guangzhou, China), which contained 65% silicon dioxide and 30% plant essential oils. The composition of plant essential oil was analysis by GC/MS (Agilent 5973 Network Mass Selective Detector, Agilent Technologies,USA), the relative content of the active components were 78.3% Cinnamic dehyde (RT = 33.6), 4% Isophorone (RT = 22.9), and eugenol (2.7%) (Fig. 5).

### Animals, treatment, management, and ethics statement

One-day-old male Cobb 500 chickens (234) were randomly divided into three groups, and each group was replicated six times with 12 chickens in each replicate. Each group was fed with: (1) a basal diet (CT), without any additives; (2) a basal diet with 400 mg/kg (PEO); (3) a basal diet with 30 mg/kg virginiamycin (VIRG). Virginiamycin purchased from Phibro Animal Health Corporation (USA). The corn-soybean-based basal diet was used, and the nutritional level of the diet was prepared following the NRC (1994) feeding standards. All experiments were performed in accordance with the approved guidelines and regulations. The diet composition and nutrient levels are shown in Table 3.

**Table 3:** Dietary compositions and nutrient levels of broilers (as-fed basis).

Ingredient (%)	(d 1–14)	(d 15–28)	(d 29–42)
Corn	55.00	57.65	59.03
Soybean meal	38.00	34.87	33.80
Soybean oil	3.00	3.50	4.00
Dicalcium phosphate	1.85	1.60	1.70
limestone	1.17	1.20	1.38
salt	0.35	0.35	0.30
Vitamin premix^a^	0.03	0.03	0.02
Choline chloride (50%)	0.10	0.07	0.05
Mineral premix^b^	0.20	0.20	0.20
DL-Met	0.25	0.16	0.07
HCl-Lys	0.05	0.04	0.03
total	100	100	100
Calculated nutrition levels			
ME (MJ/Kg)	12.16	12.78	12.82
CP (%)	20.74	19.60	18.00
Met+Cys (%)	0.92	1.05	0.64
Lys (%)	1.1	0.95	0.9
Ca (%)	1.08	0.95	0.9
P (%)	0.71	0.64	0.55

^a^Vitamin premix containing the following content, per kilogram of diet: vitamin A, 10,000 IU; vitamin D_3_(cholecalciferol), 3500 IU; vitamin E (DL-α-tocopheryl acetate), 60 mg; vitamin K (menadione), 3 mg; thiamine, 3 mg; riboflavin, 6 mg; pyridoxine, 5 mg; vitamin B_12_ (cyanocobalamin), 0.01 mg; niacin, 45 mg; pantothenic acid (D-calcium pantothenate), 11 mg; folic acid, 1 mg; biotin, 0.15 mg; choline chloride, 500 mg; ethoxyquin (antioxidant), 150 mg.

^b^Mineral premix containing the following content, per kilogram of diet: Fe, 60 mg; Mn, 100 mg; Zn, 60 mg; Cu, 10 mg; I, 1 mg; Co, 0.2 mg; Se, 0.15 mg.

Chickens were reared in a coop (100 cm × 80 cm) with two water nipples per pen. Chickens were raised routinely and immunized according to the normal immunization procedures. The diet was available ad libitum throughout the trial period. The temperature of the house was maintained at the optimum temperature of broiler chickens at 33–34 °C for the first week, and decreased from the second week by 3 °C per week until it reached 22 °C. All experimental protocols were approved by the China Agricultural University Animal Care Committee (permit number SYXK20171208).

### Sampling collection

At 28 days of age, six chickens per treatment group (one chicken per repeat) were randomly selected to collected blood from the left brachial vein. After blood was collected, the chicken was injected intravenously pentobarbital sodium (30 mg/kg body weight) and cervical dislocation was executed about 2 g of cecal digestive fluid was collected and placed in two sterilizing tubes, immediately frozen in liquid nitrogen and stored at −80 °C. One sample was used for DNA extraction and Pyrosequencing, and second one for global metabolomic analysis. The blood samples were centrifuged for 15 min (1,500 g, 4 °C). serum sample was stored at −80 °C for UPLC-Q-TOF/MS analysis.

### DNA Extraction and 16S rDNA Amplicon Pyrosequencing

Total bacterial genomic DNA was extracted from each digested sample using the Fast DNA SPIN extraction kits (MP Biomedicals, Santa Ana, CA, USA). DNA extracts were stored at −20 °C prior to further analysis. Extracted DNAs were measured using a NanoDrop ND-1000 spectrophotometer (Thermo Fisher Scientific, Waltham, MA, USA) and agarose gel electrophoresis.

PCR amplification was performed according to the method of Jinfeng Song^59^. The V3-V4 region of 16S rRNA genes was amplified using the 338F-806R primer set (338F: 55′-ACTCCTACGGGAGGCAGCA-3′. 806R:5′-GGACTACHVGGGTWTCTAAT-3′). The sample specificity 7-bp barcode was added to the primer for multiple sequencing. Thermal cycle included initial denaturation of 98 °C for 2 min, followed by 25 cycles consisting of denaturation of 98 °C for 15 s, annealing at 55 °C for 30 s, extension of 72 °C for 30 s, and final extension of 72 °C for 5 min. PCR amplifiers were purified using Agencourt AMPure Beads (Beckman Coulter, Indianapolis, IN, USA) and quantified using PicoGreen dsDNA Assay Kit (Invitrogen, Carlsbad, CA, USA)^59^. After the individual sample was quantified, the amplicon were collected in equal amount, and pair-end 2×300 bp sequencing was performed using the Illlumina MiSeq platform with MiSeq Reagent Kit v3 at Shanghai Personal Biotechnology Co., Ltd (Shanghai, China)^5^.

### Sequence analysis

The sequencing data were processed using the Quantitative Insights Into Microbial Ecology (QIIME, v1.8.0) pipeline, following Caporaso *et al*. (2010)^60^. Briefly, raw sequencing reads with complete barcode matches were assigned to the appropriate sample and identified as valid. Sequences < 150 bp long, with average Phred scores < 20, containing ambiguous bases, or with mononucleotide repeats longer than 8 bp were considered low-quality and were excluded from further analysis^61^. Paired-end reads were assembled using FLASH^62^. After chimera detection, UCLUST^63^ was used to group the remaining high-quality sequences into OTUs based on a minimum sequence identity of 97%. A representative sequence was identified for each OTU using default parameters. The OTUs were taxonomically classified using BLAST; each representative sequence was searched against the Greengenes Database^64^ and the best hit was selected^65^. An OTU table was used to record the abundance and taxonomic affiliation membership of every OTU in each sample. OTUs representing less than 0.001% of all of the sequences across all of the samples were ignored. To maintain a constant sequencing depth across all of the samples, an averaged, rounded, rarefied OTU table was generated by re-sampling an average of 100 OTU subsets at a minimum sequencing depth of 90%. This averaged OTU table was used for all of the subsequent analyses. Bioinformatics analyses of the sequence data were primarily performed in QIIME and R. v3.2.0. Raw reads were denoised and then cleaned to remove chimeras and low-quality sequences. OTU-level alpha diversity indices, such as Shannon diversity index, Chao1 richness estimate, and observed species richness were calculated based on the OTU table in QIIME, and OTU-level rank abundance curves were generated to compare the evenness and richness of OTUs among samples. Principal component analysis (PCA) was performed at the genus level^13^. LEfSe (Linear discriminant analysis effect size) was performed to detect differential abundant taxa across groups using the default parameters^66^. Operational taxonomic units were clustered with a 97% similarity threshold. Alpha diversity analysis included Shannon diversity index, Chao1 richness estimate, and observed species richness. Principal component analysis (PCA) was performed at the genus level^13^. LEfSe (Linear discriminant analysis effect size) was performed to detect differential abundant taxa across groups using the default parameters^66^.

### Sample preparation for LC-MS/MS analysis

The serum (100 μL) was added to 400 μL of ice-chilled methanol/acetonitrile (1:1, v/v) and centrifuged for 20 min (14,000 g, 4 °C) to remove the protein. The supernatant was dried in a vacuum centrifuge. For LC-MS analysis^67^, the samples were re-dissolved in 100 μL of acetonitrile/water (1:1, v/v). The same procedure was conducted using 100 mg sample from frozen cecal contents. One milliliter of cold methanol/acetonitrile (1:1, v/v) was added to each sample and mixed. The mixture was separated by centrifugation for 15 min (14,000 g, 4 °C). The supernatant was dried under vacuum centrifuge. Through the vortex dissolved in 100 μL acetonitrile and water (1:1, v/v), and under the 49 °C to 12,000 g centrifugal for 15 minutes. Monitor the stability and repeatability of the instrument analysis, quality control (QC) samples from the pool by 10 μL per sample and analysis along with other samples. Every 6 samples were periodically inserted into QC samples for analysis^68^.

### LC-MS/MS Analysis for serum and cecal contents

Determination of cecal contents and serum by using a UHPLC (1290 Infinity LC, Agilent Technologies) coupled to a quadrupole time-of-flight (AB Sciex TripleTOF 6600) in Shanghai Applied Protein Technology Co., Ltd. According to Wenqiang Fan’s method, the HILIC separation was carried out samples were analyzed by using a 2.1 mm × 100 mm ACQUIY UPLC BEH 1.7 µm column (waters, Ireland)^69^. In the ESI positive and negative mode, the mobile phase contained A = 25 mM ammonium acetate and 25 mM aqueous ammonium hydroxide, B = acetonitrile. The gradient was 85% B for 1 minute, followed by a linear drop to 65% in 11 minutes, then a drop to 40% in 0.1 minutes for 4 minutes, and ultimately increased to 85% in 0.1 minutes for 5 minutes with a rebalancing period.

ESI conditions were set as follows: the ion source Gas1 (Gas1) was 60, the ion source Gas2 (Gas2) was 60, the curtain gas (CUR) was 30, the source temperature was 600 °C, and the ion spray voltage fluctuation (ISVF) was ±5500 V. In the MS only acquisition, the instrument was set to collect data in the range of 60–1000 Da, and the TOF MS scanning accumulation time was set to 0.20 s/spectrum. In the automatic MS/MS acquisition, the instrument was set at the range of m/z and above 25–1000 Da, and the production scanning accumulation time was set at 0.05 s/spectrum^70^. The production scan is acquired using information dependent acquisition (IDA) with the selection of high sensitivity mode. The parameters were set as follows^71^: the collision energy (CE) was fixed at 35 V with ±15 eV; declustering potential (DP), 60 V(+) and −60V(−); exclude isotopes within 4 Da; and 10 candidate ions to monitor per cycle.

The raw MS data (wiff. scan files) were converted to MzXML files using ProteoWizard MSConvert before importing it into freely available XCMS software^67^. For peak selection, the following parameters were used: centWave m/z= 25 ppm, peak width = c (10, 60), prefilter = c (10, 100). For peak grouping, bw = 5, mzwid = 0.025, minfrac = 0.5 were used^71^. CAMERA (Collection of Algorithms of MEtabolite pRofile Annotation) was used for annotation of isotopes and admixtures. In the extracted ion features, only more than 50% of the variables of at least one set of non-zero measurements were retained. The variation coefficient (CV) of metabolites in the QC samples was set at a threshold value of 30%, which was used as the standard for evaluating the repeatability of the metabolomics data set. Compound identification of metabolites was performed by comparing the accuracy of m/z value (<25 ppm), and MS/MS spectra with an in-house database established using reliable standards^72^.

### Statistical analysis

After normalizing the processed data to the total peak intensity, it was uploaded before importing into SIMCA-P (version 14.1, Umetrics, Umea, Sweden), for multivariate data analysis, including pareto scale principal component analysis (PCA) and orthogonal partial least squares discriminant analysis (OPLS-DA)^71^. The importance of each variable in the OPLS-DA model in the projection (VIP) value was calculated to indicate its contribution to the classification. Metabolites with the VIP score > 1 were further applied to Student’s t-test at univariate level to measure the significance of each metabolite, the P values less than 0.05 were considered as statistically significant. The correlative analysis between different cecal microbial species and microbial or serum metabolites was used for calculating by Spearman’s correlations coefficient^13^.

## Supplementary information

Supplementary Table 1.

Supplementary Table 2.
